# Risk Factors for Non-alcoholic Fatty Liver Disease from the Perspective of Medical Professionals: A Systematic Review and Expert Opinion

**DOI:** 10.34172/mejdd.2025.408

**Published:** 2025-01-31

**Authors:** Mahdie ShojaeiBaghini, Mohammadreza Fattahi, Mehdi Mohammadi, Fateme Hamzavi

**Affiliations:** ^1^Medical Informatics Research Center, Institute for Futures Studies in Health, Kerman University of Medical Sciences, Kerman, Iran; ^2^Gastroenterohepatology Research Center, Shiraz University of Medical Sciences, Shiraz, Iran; ^3^Health Information Department, School of Management and Medical Informatics, Kerman University of Medical Science, Kerman, Iran

**Keywords:** Non-alcoholic fatty liver disease, Risk factor, Expert panel, Metabolic syndrome

## Abstract

**Background:**

The incidence of non-alcoholic fatty liver disease (NAFLD) has increased with the global increase in the outbreak of obesity, type 2 diabetes, and metabolic syndrome, reaching a 25% prevalence. However, there is currently no effective treatment for this disease apart from lifestyle modification. Furthermore, NAFLD commonly presents without symptoms, hence, leading to potentially severe and irreparable consequences.

**Methods:**

This study was based on a systematic review. The search used the keywords "non-alcoholic fatty liver" and "risk factor" across the PubMed, Scopus, and Web of Science databases. First, the articles were evaluated based on their abstract and then on their full text. The risk factors were extracted from the articles and entered into the Excel form, and then a dataset was provided to the expert panel. The risk factors were investigated, and those related to NAFLD were selected.

**Results:**

The results led to the identification of 180 risk factors in 15 categories. First, the risk factors mentioned in fewer than five articles were removed. Then, the remaining 101 risk factors were presented to the expert panel, of which 39 risk factors related to NAFLD were selected.

**Conclusion:**

In summary, this study shows that NAFLD is caused by various factors such as metabolic syndrome, certain diseases, demographic information, specific surgeries, drug consumption, different foods and beverages, occupation, physical activity status, and socioeconomic status. Recognizing these risk factors enables doctors to make earlier diagnoses, potentially preventing disease progression. Additionally, it is possible to develop treatment strategies aimed at reducing the risk factors of the disease, which could result in fewer patients suffering from NAFLD in the future.

## Introduction

 Non-alcoholic fatty liver disease (NAFLD) is a type of liver disease characterized by the abnormal accumulation of fat in the liver and insulin resistance. In individuals with NAFLD, the triglyceride accumulation in hepatocytes exceeds 5% of liver weight, while in healthy people, this amount is approximately 1.9%. In the general population, it is around 3.9%.^1^ The global increase in obesity, type 2 diabetes, and metabolic syndrome h0as contributed to the rising incidence of NAFLD,^2^ with the prevalence of this disease reaching 25.2% worldwide.^3^ Clinically, the progression of NAFLD is divided into five stages: non-alcoholic steatosis, non-alcoholic steatohepatitis, liver fibrosis, liver cirrhosis, and liver carcinoma. Furthermore, the occurrence of NAFLD and liver fibrosis is closely related to an increase in mortality from liver-related diseases.^4^

 Currently, there is no effective cure for NAFLD other than lifestyle changes, which include limiting energy intake and increasing physical activity.^5^ The outcomes of NAFLD are varied and can range from progression to chronic liver disease with associated complications and mortality to worsening insulin resistance and type 2 diabetes, as well as contributing cardiovascular disease and chronic kidney disease. Therefore, NAFLD presents a complex problem with consequences beyond the liver.^6^ Approximately 48% to 100% of patients with NAFLD are asymptomatic. Diagnosis often follows abnormal findings from routine biochemistry tests, abdominal ultrasounds, or cardiovascular risk assessments. Screening for NAFLD in asymptomatic patients, as well as in high-risk patients, is controversial.^7^ It has also been shown that the economic burden of NAFLD is significant worldwide. In the United States, more than 64 million people are predicted to be affected by NAFLD, with yearly direct medical costs estimated at $103 billion. More than 52 million people in Europe are affected, with annual costs of around $35 billion.^8^ Previous research has indicated that individuals with NAFLD often report a lack of information about their diagnosis, perceive NAFLD as a relatively benign disease, and experience gaps in knowledge regarding lifestyle modifications as the primary treatment for the condition. Limitations to exercise include a lack of resources and training, physical discomfort, and time constraints. Additionally, barriers to maintaining healthy eating patterns among individuals with NAFLD include time constraints, life stressors, and the need for convenience.^9^ Therefore, this study aims to identify the risk factors associated with this disease to facilitate its diagnosis.

## Materials and Methods

**Figure 1:**
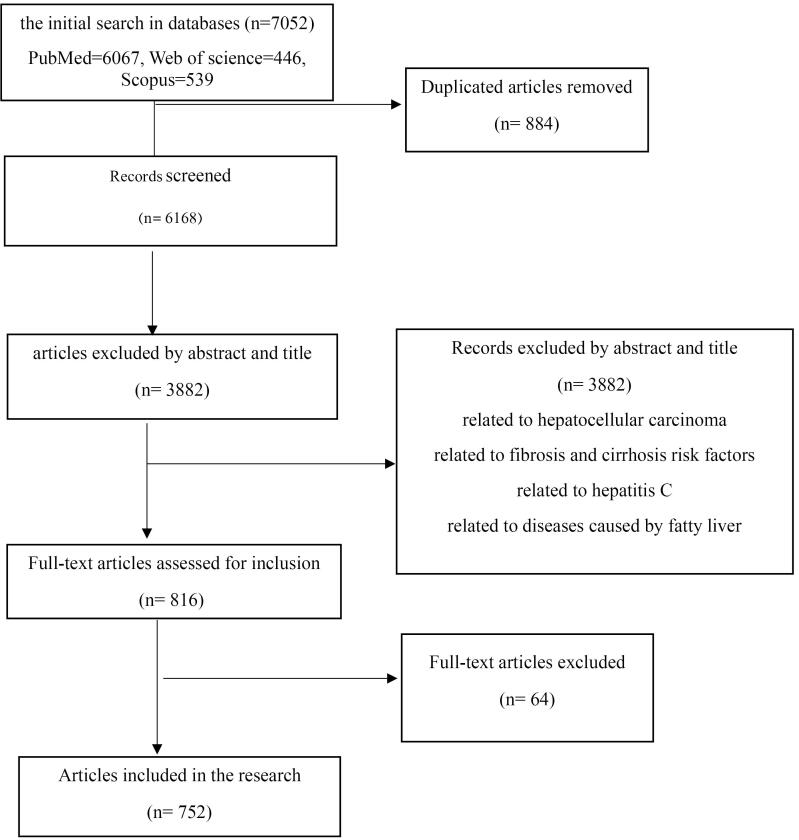
Flow diagram of the selected articles

 This systematic review distinguished the risk factors for NAFLD. The study was conducted based on a review of databases and a comprehensive library review. The keywords “non-alcoholic fatty liver disease” and “risk factor” were searched in MeSH. The search encompassed related articles in PubMed, Scopus, and Web of Science databases up to November 2023. The criteria for inclusion and exclusion of articles in the study included relevance to the research topic, access to the full text, and publication in Persian or English. Initially, articles from all years were selected; after removing irrelevant items based on their abstract, articles published between 2020 and 2023 were chosen for full-text evaluation. Risk factors were extracted from the mentioned articles and entered into an Excel spreadsheet. A PRISMA flow diagram was used to collect data, as shown in Figure 1.

 The extracted risk factors were compiled into a dataset and discussed at an expert panel meeting consisting of six liver and gastroenterology doctors. The criteria for experts to participate included having at least five years of experience in NAFLD and a willingness to engage in the study. Risk factors that appeared in more than five articles were examined, and each risk factor related to NAFLD was selected.

## Results

 The review results led to the collecting of 180 risk factors categorized into 15 sections. These include metabolic syndrome, liver enzymes, diseases, hormones, laboratory tests, demographic information, surgeries, medications, dietary factors, vitamins, pregnancy, exposure, menstruation, occupation, and other relevant factors. A detailed list of the data elements according to these sections is presented in Table 1.

**Table 1:** Risk factors extracted from articles

**Parts**	**Risk factors**
Metabolic syndrome	Obesity, diabetes, dyslipidemia, hypertension
Liver enzymes	Alanine aminotransferase, aspartate aminotransferase, alkaline phosphatase, gamma glutamyl transpeptidase, albumin, bilirubin
Diseases	Polycystic ovarian syndrome, obstructive sleep apnea, hypothyroidism, pancreatic cancer, gallstone disease, hyperaldosteronism, atopic dermatitis, breast cancer, hidradenitis suppurativa, colonic diverticulosis, kidney disease, stress, gout, toxoplasmosis, celiac, Wilson, Weber-Christian, phenylketonuria, Maple syrup urine disease, Hemophilia, Turner syndrome, Cushing’s syndrome, inflammatory bowel disease, hypo pituitary, psoriasis, periodontitis, apoptosis, sarcopenia, mental disorder, lipodystrophies, gut microbiota, helicobacter pylori, Epstein bar virus, hepatitis C virus, hepatitis B virus, Human immunodeficiency virus
Hormones	Sex hormone-binding globulin (SHBG), estradiol, estrogen, follicle-stimulating hormone, thyroid-stimulating hormone, T3, T4, glucagon, prolactin, growth hormone deficiency, adipokines, hepatokines, transforming growth factor-β, pro-inflammatory cytokines
Lab tests	Ferritin, mean platelet volume, red blood cell, white blood cell, afamin, C-reactive protein (hs-CRP), micronutrient, cystatin c (CysC), bile acids, creatinine, uric acid, complement C3, anti-thyroid peroxidase antibody (TPOAb), urinary retinol-binding protein/creatinine ratio, plasma free fatty acid (FFA), osteopontin (OPN), lipoprotein, succinate receptor 1 (SUCNR1), bone morphogenetic protein-9 (BMP-9), lipocalin-type prostaglandin synthase (L-PGDS), angiopoietin-like proteins (ANGPTLs), dipeptidyl-peptidase 4 (DPP4), plasminogen activator inhibitor-1
Demographic information	Age, sex, family history, racial, genetics, smoking, cigarette cessation, alcohol
Surgeries	Liver transplantation, oophorectomy, hysterectomy, cholecystectomy, abortion, bowel surgery, pancreatic resection
Drugs	Amiodarone, tamoxifen, methotrexate, glucocorticosteroids, cocaine, thiopurines, hormone replacement therapy (HRT), antipsychotic, antibiotics, sulfonylurea, contraceptives, nonsteroidal anti-inflammatory, amino salicylates, valproic acid, antiretroviral treatment, trimethylamine-N-oxide (TMAO), exogenous hormone use
Dietary factors	Egg, fried foods, nuts, pickles, sweets, salty (sodium) foods, smoked foods, potatoes, wheat flour, sauces, ultra-processed food, fast foods, high-fat diet, red meat, processed meat, organ meat, chicken, soft drink, tea, carbohydrate, sugar, gluten-free diet, fatty acid, high-fat dairy products, refine grains, breastfeeding, advanced glycation end products (AGEs), dietary inflammatory index (DII)
Vitamins	Vit A, Vit B, Vit C, Vit E
Gestation	GDM, pregnancy-induced hypertension, pre-eclampsia (PE), cesarean section, maternal obesity or overnutrition
Exposure to	Metals, particulate matter (PM2.5), industrial chemicals, polycyclic aromatic hydrocarbons (PAHs), organic solvents, light pollution
Menstruation	Post menopause, early menarche
Occupation	Shift workers, Job
Other factors	Less physical activity, rapid weight loss, oxidative stress, endoplasmic reticulum stress, eating fast, socioeconomic status, phlegm-dampness, blood-stasis, toothbrushing, syndecan-4 (SDC4), lantibiotic, mitochondrial dysfunction, endotoxin, heart rate variability (HRV), autonomic nervous system, disruption in circadian rhythm, > 2 hours of screen time per day

 After collecting the risk factors from the articles and entering them into Excel, we removed those that were repeated in fewer than five articles, except for cholecystectomy, wheat flour, potatoes, and vitamin A, based on the opinion of a liver and gastroenterology expert. Ultimately, 79 risk factors were excluded, and 101 were included in the expert panel survey. These elements are listed by section in Table 2.

**Table 2:** Risk factors repeated in less than five articles

**Parts**	**Risk factors**
Diseases	pancreatic cancer, gallstone disease, hyperaldosteronism, atopic dermatitis, breast cancer, hidradenitis suppurativa, colonic diverticulosis, kidney disease, stress, gout, toxoplasmosis, celiac, Wilson, Weber-Christian, phenylketonuria, Maple syrup urine disease, hemophilia, Turner syndrome, Cushing’s syndrome, Epstein bar virus
Hormones	Follicle-stimulating hormone, glucagon, prolactin, growth hormone deficiency
Lab tests	Osteopontin (OPN), lipoprotein, cystatin c (CysC), complement C3, anti-thyroid peroxidase antibody (TPOAb), urinary RBP/creatinine ratio, succinate receptor 1 (SUCNR1), bone morphogenetic protein-9 (BMP-9), lipocalin-type prostaglandin synthase (L-PGDS), angiopoietin-like proteins (ANGPTLs), dipeptidyl-peptidase 4 (DPP4), plasminogen activator inhibitor-1
Demographic information	Cigarette cessation
Surgeries	Oophorectomy, hysterectomy, abortion, bowel surgery, pancreatic resection
Drugs	Cocaine, thiopurines, hormone replacement therapy (HRT), sulfonylurea, contraceptives, amino salicylates, valproic acid, antiretroviral treatment, trimethylamine-N-oxide (TMAO), exogenous hormone use
Dietary factors	Nuts, pickles, smoked foods, ultra-processed food, tea, gluten-free diet, breastfeeding, advanced glycation end products (AGEs), dietary inflammatory index (DII)
Vitamins	Vit C, Vit E
Gestation	Pregnancy-induced hypertension, pre-eclampsia (PE), cesarean section
Exposure to	Polycyclic aromatic hydrocarbons (PAHs), organic solvents, light pollution
Other factors	Rapid weight loss, eating fast, phlegm-dampness, blood-stasis, toothbrushing, Syndecan-4 (SDC4), lantibiotic, heart rate variability (HRV), autonomic nervous system, > 2 hours of screen time per day

 Finally, 101 risk factors were presented to the experts as a dataset, and the relationship of each risk factor with NAFLD was discussed and investigated. As a result, the relationship of 39 risk factors with NAFLD was confirmed. These factors are listed separately in Table 3.

**Table 3:** Risk factors related to NAFLD confirmed by experts

**Parts **	**Risk factors**
Metabolic syndrome	Obesity, diabetes, dyslipidemia, hypertension
Diseases	Polycystic ovarian syndrome, obstructive sleep apnea, hypothyroidism, hepatitis c virus, gut microbiota
Demographic information	Age, sex, family history, racial, genetics, alcohol
Surgeries	Liver transplantation, cholecystectomy
Drugs	Amiodarone, tamoxifen, methotrexate, glucocorticosteroids
Dietary factors	Fried foods, sweets, potatoes, wheat flour, refine grain, fast foods, high-fat diet, red meat, processed meat, chicken, soft drink, carbohydrate, sugar, fatty acid, high-fat dairy products
Occupation	Job
Other factors	Less physical activity, socioeconomic status

## Discussion

 This research aimed to distinguish the risk factors of NAFLD from the perspectives of experts. Understanding the risk factors associated with NAFLD is essential for preventing this disease, which has been increasing in recent years due to lifestyle changes.

 Based on the results of a comprehensive review of previous studies and a survey of specialist doctors to identify the risk factors of NAFLD, these factors were classified into eight categories. Various factors, such as metabolic syndrome, certain diseases, demographic information, specific surgeries, the consumption of certain medications, dietary habits, occupation, activity status, and social factors, can influence the development of this disease.

 Comprehensive studies have shown that metabolic syndrome, which includes obesity, type 2 diabetes, dyslipidemia, and hypertension, is one of the leading causes of NAFLD. According to Chang et al, in their observational and cross-sectional study, which involved 706 nurses, the results demonstrated a correlation between metabolic syndrome and NAFLD. Nurses who were older, had a higher body mass index (BMI), waist circumference beyond the standard range, fasting blood glucose levels between 100-125 mg/dL, and very low HDL-C levels were identified as being at risk for NAFLD.^10^ Bagheri Lankarani et al conducted a cross-sectional study titled “Non-alcoholic fatty liver disease in southern Iran: a population-based study,” which involved 819 participants. The results indicated that NAFLD was associated with age, BMI, hypertension, blood sugar levels, high cholesterol, and triglycerides, highlighting the relationship between metabolic syndrome and NAFLD^11^ Most of the findings in our research were related to obesity, diabetes, dyslipidemia, and hypertension, with 599, 484, 437, and 246 occurrences, respectively.

 Despite the studies demonstrating the relationship between obesity and NAFLD, some research has acknowledged that NAFLD can occur in individuals without obesity risk factors, a condition referred to as lean NAFLD.^12-14^ Additionally, studies have shown that higher rates of diabetes, central obesity, and dyslipidemia are observed in patients with polycystic ovarian syndrome.^15^ Hypothyroidism and obstructive sleep apnea have also been linked to an increased incidence of NAFLD. In a study by Paschou and colleagues, the prevalence of NAFLD in women with polycystic ovary syndrome was 34%-70%, compared with 14%-34% in healthy women.^16^ Similarly, the study by Augustine and colleagues demonstrated a significant relationship between dyslipidemia and TSH levels, concluding that hypothyroidism was one of the contributing factors to NAFLD.^17^ In the study by Zhang and colleagues, titled “The effect of obstructive sleep apnea on fatty liver disease may be obscured by alcohol consumption: An ordinal logistic regression analysis”, it was stated that obstructive sleep apnea is a determining factor for the advancement of NAFLD.^18^

 Several studies indicate that genetic and environmental factors, such as diet and physical activity, play a central role in the incidence of NAFLD.^19-21^ Understanding the impact of nutritional, lifestyle, and metabolic factors on NAFLD and increasing public awareness is essential.^22^ Several studies have revealed that high consumption of red meat, high-fat dairy products, fast foods, soft drinks, processed meat, refined grains, sweets, and carbohydrates increases the incidence of NAFLD.^23-25^ These findings are consistent with the opinions of expert doctors in the present study.

 Aging and reduced physical activity expose many individuals to the risk of NAFLD, a concern mentioned in numerous studies. Research has shown that a higher level of physical activity is associated with decreased fat accumulation in the liver. Moreover, it has been acknowledged that the prevalence of NAFLD grows with aging, necessitating urgent and comprehensive measures to address this increasing burden.^26-29^

 Limitations of the present study include the fact that we could only include articles in Persian and English, but there were still articles in the field of study that were in other languages. Although we used three databases to search articles, there might be related articles in different databases. Finally, there may be an article related to our research, but it has not been published.

## Conclusion

 In summary, the results of this study indicate that NAFLD is influenced by various factors, including metabolic syndrome, certain diseases, demographic information, surgical history, drug consumption, dietary habits, occupational factors, physical activity, and socioeconomic status. Understanding these factors enables doctors to diagnose this disease earlier and helps prevent its progression. Moreover, advancing treatment strategies to mitigate the risk factors associated with NAFLD could lead to a plummet in the number of patients affected by this condition in the future.
